# Changes and Appointments in Medical Schools

**Published:** 1860-11

**Authors:** 


CHANGES AND APPOINTMENTS IN MEDICAL SCHOOLS.—The Medical Department of the *University of Michigan* has sustained a loss in the death of Dr. Samuel Denton, Professor of Theory and Practice, and of Pathology. The chair thus rendered vacant has been filled by the election of Dr. A. B. Palmer, transferred from the professorship of Materia Medica, Therapeutics, and Diseases of Women and Children, in the same school. The last-mentioned chair remains still vacant, and its duties have been distributed among the members of the faculty. The new Professor of Practice and Pathology has but recently returned from a tour in Europe, and is well and favorably known to his brethren throughout the United States by his editorship of one of the most useful journals in the country.

*New York Medical College.*—This institution has been reorganized, Professors Carnochan and Doremus being the only two of the old faculty connected with it. The other professorships are constituted as follows: D. M. Reese, M.D., Medicine and Medical Jurisprudence; B. J. Raphael, M.D., Principles of Surgery and Surgical Pathology; A. K. Gardner, M.D., Clinical Midwifery and Diseases of Women; J. D. Bronson, M.D., Anatomy; C. A. Budd, M.D., Theory and Practice of Midwifery; A. Jacobi, M.D., Infantile Pathology and Therapeutics; B. L. Budd, M.D., Toxicology; R. K. Browne, Physiology. The chairs of Materia Medica and Clinical Medicine still remain vacant.

*Shelby Medical College.*—Professor Maddin has been transferred from the chair of Anatomy to that of Surgery, vacated by Dr. May; and Dr. D. B. Cliffe has been appointed to the professorship of Anatomy. Dr. Haskins is succeeded in the chair of Practice of Physic by Dr. J. J. Abernathy.

*Lind University.*—Dr. Deville has vacated the chair of Anatomy, which has been filled by the appointment of Dr. J. Hollister; and Dr. A. L McArthur has been created Professor of Materia Medica and Therapeutics.

*University of Buffalo.*—Dr. E. M. Moore succeeds Professor Hamilton in the chair of Surgery, and Dr. Wm. Mason has been elected to that of Physiology.

Dr. G. M. B. Maughs has been called to the chair of Physiology and Chemistry in the Medical Department of the *Missouri State University.*

Dr. J. T. Maxwell has accepted the Professorship of Obstetrics in the *Oglethorpe Medical College,* and Dr. J. H. Tate fills the same position in the *Cincinnati College of Medicine and Surgery.*

Professor Lawson has received the appointment to the chair of Clinical Medicine in the Medical Department of the *University of Louisiana.*

